# Supplementary material for: Impact of Endoluminal Radiofrequency Ablation on Immunity in Pancreatic Cancer and Cholangiocarcinoma
Source: Biomedicines. 2022 Jun 6;10(6):1331. doi: 10.3390/biomedicines10061331 (PMC9219740; doi:10.3390/biomedicines10061331)
Supplement: Supplementary file 1 [file biomedicines-10-01331-s001.zip › biomedicines-1751539-supplementary.pdf]

Supplementary Table S1. Association between cytokine overexpression and survival.

PDAC	AG			UG			
	NOC	NOE	MS	NOC	NOE	MS	p*
IL-1α	6	1	N/A	143	81	19.58	0.0925
IL-1β	11	4	34.79	138	78	19.48	0.250
IL-6	12	5	49.34	137	77	19.65	0.603
IL-18	14	9	15.11	135	73	19.81	0.796
IL-33	10	6	43.76	139	76	19.65	0.530
IL-36β	10	4	19.81	139	78	19.58	0.460
TNFα	14	9	22.70	135	73	19.65	0.836
IL-1RA	6	2	20.17	143	80	19.65	0.946
IL-10	14	8	16.98	135	74	19.81	0.483
CXCL1	8	4	12.91	141	78	19.81	0.794
CXCL5	11	8	12.94	138	74	19.65	0.425
CXCL8	12	9	10.12	137	73	19.94	0.248
**CXCL9**	10	10	**8.77**	139	72	**20.17**	**6.145e-4**
**CXCL10**	13	12	**9.82**	136	70	**20.17**	**9.738e-4**
**CXCL11**	14	12	**8.77**	135	70	**20.60**	**4.401e-4**
CCL21	9	4	17.90	140	78	19.65	0.999
CCL2	9	4	24.05	140	78	19.58	0.611
CCL5	9	6	16.98	140	76	19.94	0.0774

CCC	AG			UG			
	NOC	NOE	MS	NOC	NOE	MS	p*
IL-1α	4	0	N/A	29	15	24.34	0.209
IL-1β	1	0	N/A	32	15	24.34	0.624
IL-6	3	1	12.65	30	14	24.34	0.612
IL-18	1	1	63.70	32	14	24.34	0.595
IL-33	3	2	11.14	30	13	30.35	0.0985
IL-36β	5	1	N/A	28	14	24.34	0.315
TNFα	4	1	23.03	29	14	24.34	0.495
IL-1RA	2	1	24.34	31	14	30.35	0.824
IL-10	5	1	N/A	28	14	23.03	0.446
CXCL1	2	1	0.85	31	14	24.34	0.910
CXCL5	3	2	**8.90**	30	13	**30.35**	**0.0215**
CXCL8	3	0	N/A	30	15	24.34	0.432
CXCL9	2	0	N/A	31	15	24.34	0.222
CXCL10	3	0	N/A	30	15	24.34	0.183
CXCL11	4	0	N/A	29	15	24.34	0.162
CCL21	3	0	N/A	30	15	23.03	0.233
CCL2	5	1	12.65	28	14	24.34	0.719
CCL5	3	1	N/A	30	14	24.34	0.663

PDAC, pancreatic ductual adenocarcinoma; CCC, cholangiocarcinoma; AG, altered group (putative copy-number alterations; mRNA expression z-score of 1.5 relative to diploid samples); UG, unaltered group; NOC, number of cases (total); NOE, number of Events; MS, median survival (months); p, logrank test p-value

dataset: The Cancer Genome Atlas; tool: cBioPortal

Supplementary Table S2. Association between cytokine expression and tumor immune cell infiltration.

PDAC	II	algorithm	COR	Rho	p
CXCL1	neutrophil	TIMER	**positive**	0.39	**1.31e-07**
CXCL5	neutrophil	TIMER	**positive**	0.331	**9.53e-06**
CXCL8	N/A				
CXCL9	T cell CD8+	TIMER	**positive**	0.609	**9.11e-19**
	T cell CD4+	TIMER	**positive**	0.271	**3.39e-04**
CXCL10	T cell CD8+	TIMER	**positive**	0.461	**2.14e-10**
	T cell CD4+	TIMER	NS	0.007	9.27e-01
CXCL11	T cell CD8+	TIMER	**positive**	0.330	**1.06e-05**
	T cell CD4+	TIMER	NS	0.026	7.39e-01
CCL21	T cell CD8+	TIMER	**positive**	0.536	**3.99e-14**
	T cell CD4+	TIMER	**positive**	0.340	**5.29e-06**
CCL2	monocyte	XCELL	**positive**	0.411	**2.27e-08**
	NK cell	XCELL	NS	-0.093	2.28e-01
	eosinophil	XCELL	NS	-0.031	6.92e-01
CCL5	monocyte	XCELL	**positive**	0.531	**7.48e-14**
	NK cell	XCELL	NS	-0.02	7.96e-01
	eosinophil	XCELL	NS	-0.089	2.48e-01

CCC	II	algorithm	COR	Rho	p
CXCL1	neutrophil	TIMER	NS	0.105	5.49e-01
CXCL5	neutrophil	TIMER	NS	0.319	6.17e-02
CXCL8	N/A				
CXCL9	T cell CD8+	TIMER	**positive**	0.448	**6.96e-03**
	T cell CD4+	TIMER	**positive**	0.469	**4.46e-03**
CXCL10	T cell CD8+	TIMER	**positive**	0.431	**9.74e-03**
	T cell CD4+	TIMER	NS	-0.185	2.88e-01
CXCL11	T cell CD8+	TIMER	**positive**	0.509	**1.77e-03**
	T cell CD4+	TIMER	NS	0.256	1.38e-01
CCL21	T cell CD8+	TIMER	NS	0.221	2.01e-01
	T cell CD4+	TIMER	NS	-0.043	8.08e-1
CCL2	monocyte	XCELL	NS	0.08	6.49e-01
	NK cell	XCELL	NS	-0.154	3.78e-01
	eosinophil	XCELL	NS	0.156	3.71e-01
CCL5	monocyte	XCELL	NS	0.092	5.97e-01
	NK cell	XCELL	NS	0.172	3.23e-01
	eosinophil	XCELL	NS	-0.144	4.09e-01

PDAC, pancreatic ductual adenocarcinoma; CCC, cholangiocarcinoma; II, immune infiltrate; COR, correlation of gene expression and immune cells infiltration; N/A, gene expression not available in the tool; NS, not significant, p, logrank test p-value

dataset: The Cancer Genome Atlas; tool: TIMER
